# Fatty acid metabolism in glioma: a review of novel targets and therapeutic strategies

**DOI:** 10.1186/s41016-026-00442-w

**Published:** 2026-07-31

**Authors:** Xiao Zhou, Di Wang, Wei Zhang, Guanzhang Li

**Affiliations:** 1 Department of Neurosurgery, Beijing Tiantan Hospital, Capital Medical University, No.119 South Fourth Ring Rd West, Fengtai District, Beijing, 100070 People’s Republic of China; 2 Department of Molecular Neuropathology, Beijing Neurosurgical Institute, Capital Medical University, No.119 South Fourth Ring Rd West, Fengtai District, Beijing, 100070 People’s Republic of China; 3 Department of Central Laboratory, Beijing Neurosurgical Institute, Capital Medical University, Beijing, 100070 People’s Republic of China; 4 China National Clinical Research Center for Neurological Diseases, Beijing, 100070 People’s Republic of China; 5 Brain Tumor Center, Beijing Institute of Brain Disorders, Capital Medical University, Beijing, 100070 People’s Republic of China; 6 Beijing Engineering Research Center of Targeted Drugs and Cell Therapy for CNS Tumors, Beijing, 102600 People’s Republic of China; 7 Chinese Glioma Genome Atlas Network (CGGA) and Asian Glioma Genome Atlas Network (AGGA), Beijing, 100070 People’s Republic of China

**Keywords:** Glioma, Fatty Acid Metabolism, Metabolic Reprogramming, Therapeutic Resistance

## Abstract

Glioma is the most common primary malignant brain tumor in adults, among which glioblastoma (GBM) shows the highest degree of malignancy and the worst prognosis. Although standard therapeutic approaches, including surgical resection, radiotherapy, and chemotherapy, are routinely applied, the prognosis of glioma, especially GBM, remains poor. Metabolic rewiring is widely recognized as a major characteristic of malignant tumors and is especially evident in glioma. Recent studies have shown that fatty acid metabolic rewiring is a widespread feature of glioma and plays an important role in its pathogenesis and therapeutic resistance. Therefore, targeting metabolic reprogramming, particularly aberrant fatty acid metabolism, may have potential therapeutic implications in glioma. This review systematically synthesizes the molecular mechanisms underlying abnormal fatty acid metabolism in glioma pathogenesis and treatment responses. It also elucidates the regulatory networks governing key fatty acid metabolic pathways and integrates emerging strategies targeting these pathways to enhance the therapeutic sensitivity of glioma cells. Ultimately, this review aims to provide a conceptual framework and future research directions for overcoming the current limitations of glioma treatment through metabolic therapy.

## Background

In adults, glioma is the predominant primary intracranial malignant tumor. Isocitrate dehydrogenase (IDH)-wildtype glioblastoma (GBM) is characterized by the highest malignancy grade and the most unfavorable clinical prognosis [1]. The median overall survival (mOS) of GBM is 14 to 16 months [2–7]. The Stupp protocol remains the first-line standard of treatment for GBM, involving maximal safe tumor resection followed by adjuvant radiotherapy and chemotherapy [7, 8]. Although basic research on glioma has advanced considerably in recent years, the limited sensitivity of GBM to conventional radiotherapy and chemotherapy continues to hinder meaningful improvements in patient prognosis. Given these limitations, there is an urgent need in clinical practice and fundamental research to identify innovative treatment strategies in glioma. A growing body of evidence has shown that metabolic rewiring contributes to the malignant phenotypes of tumor cells, including uncontrolled proliferation and resistance to therapy. Recent study has indicated that abnormalities in fatty acid (FA) metabolism substantially contribute to glioma cell resistance to radiotherapy, chemotherapy, and immunotherapy. These findings show that modulation of fatty acid metabolism may offer a potential strategy for improving the limited therapeutic efficacy currently observed in GBM [9, 10]. Therefore, it is essential to systematically clarify the molecular mechanisms through which fatty acid metabolic rewiring affects the biological behavior of gliomas. Simultaneously, developing targeted therapies to modulate fatty acid metabolism is likely to improve outcomes in GBM patients.

Fatty acids (FAs) are fundamental molecules that are indispensable for cellular function and perform a wide range of essential physiological roles. FAs can generate adenosine triphosphate (ATP) through β-oxidation, serving as crucial energy sources or energy storage. Furthermore, FAs, as integral structural components of biomembranes, can be used to synthesize phospholipids via the diacylglycerol (DG) pathway or the cytidine diphosphate-diacylglycerol (CDP-DG) pathway, and also combine with sphingosine to form glycolipids. Phospholipids and glycolipids are essential for maintaining membrane structural integrity and supporting membrane-associated biological functions. Notably, glycolipids are also implicated in many critical physiological processes, including cell recognition, inflammatory responses, and immune modulation. In addition, certain FA derivatives, especially those derived from polyunsaturated fatty acids (PUFAs), may function as second messengers in cellular signaling pathways. For example, arachidonic acid (AA) can be metabolized through the cyclooxygenase (COX) pathway to produce prostaglandins (PGs) and thromboxane A2 (TXA2), or through the lipoxygenase (LOX) pathway to form leukotrienes, thereby regulating essential cellular events such as proliferation and differentiation.

Otto Warburg first reported in 1923 that tumor cells preferentially generate energy through glycolysis instead of oxidative phosphorylation, despite the availability of oxygen. Such a phenomenon is referred to as the Warburg Effect. Warburg proposed that abnormal metabolism in tumor cells is fundamental to their malignant proliferation. This theory laid the foundation for subsequent investigations in the area of cancer metabolism [11]. In the field of glioma treatment, although strategies targeting the Warburg Effect have shown some therapeutic potential in preclinical studies [10], the ultimate clinical value and therapeutic efficacy of these strategies still require further validation in clinical trials.

**Fig. 1:**
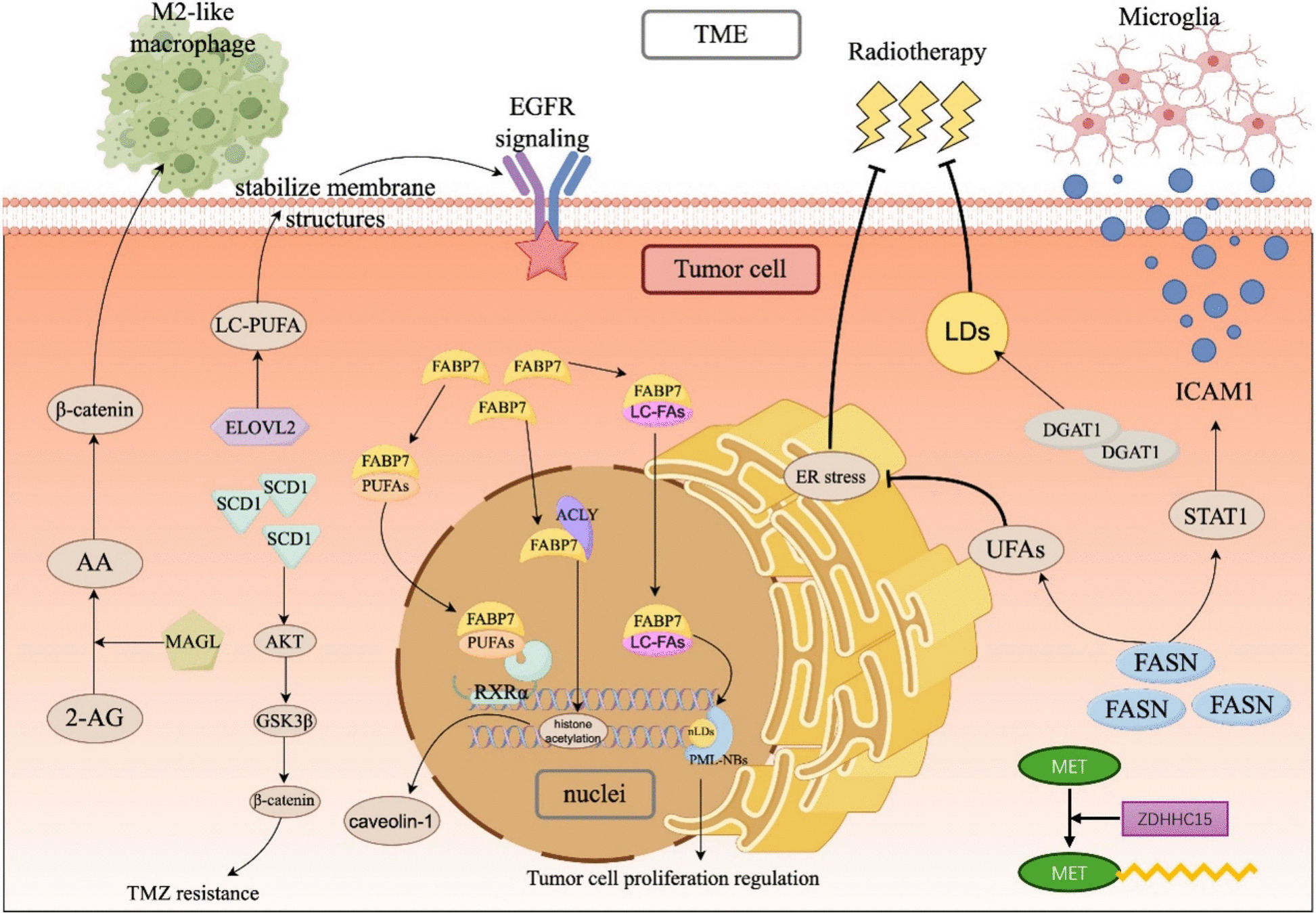
The mechanisms of FA metabolic reprogramming in glioma

In addition to impaired glucose metabolism, alterations in fatty acid metabolism constitute a prominent metabolic feature of glioma cells [9]. A better understanding of fatty acid metabolic features in glioma cells may help clarify the mechanisms underlying malignant proliferation and invasion and may also open new avenues for target discovery and anti-tumor drug development. This review aims to systematically integrate recent research progress in glioma fatty acid metabolic reprogramming (Table 1, Fig. 1). Based on current advances and major research focuses in the field, this review discusses the molecular mechanisms by which aberrant fatty acid metabolism facilitates glioma cells' proliferation, enhances the migration and invasion abilities, remodels the tumor microenvironment (TME), stimulates angiogenesis, and causes resistance to chemoradiotherapy.

**Table 1:** The mechanisms of FA metabolic reprogramming in glioma

Targets	mechanisms	FA metabolic process
FASN	1. Radiotherapy-induced excessive activation of FASN generates a large amount of UFAs, which results in resistance to radiotherapy by alleviating ER stress [12] 2. The FASN/STAT1/ICAM1/microglia axis facilitates the recruitment of microglia, thereby remodeling the TME [13]	De novo synthesis
ZDHHC	ZDHHC15-mediated palmitoylation of c-MET promotes its O-glycosylation, homodimerization, and activation, thereby maintaining the stemness of GSCs [14]	Post-translational modification
FABP7	1. FABP7 interacts with ACLY and regulates the expression of caveolin-1 through epigenetic modifications, thereby modulating the biological characteristics of tumors [15] 2. FABP7 interacts with long-chain FAs, promoting the formation of nLDs colocalized with PML-NBs and driving tumor cell proliferation [16] 3. PUFAs can be translocated to the nucleus by binding to FABP7, triggering the activation of the nuclear receptor RXRα, and inducing the stationary-to-migratory transition of GSCs [17]	Transport
SCD1	1. D-2HG in IDH1-mutant gliomas can upregulate the expression of SCD1, increase intracellular MUFA, and cause the expansion of the Golgi apparatus and endoplasmic reticulum [18] 2. SCD1 can mediate TMZ resistance by activating the Akt/GSK3β/β-catenin signaling pathway [19]	Desaturation and membrane remodeling
DGAT1	DGAT1 facilitates LD biogenesis to sustain GBM lipid homeostasis and mediate radiotherapy resistance [20]	Storage
MAGL	MAGL can catalyze 2-AG to AA and promote M2-like macrophage polarization through the MAGL/PGE₂/β-catenin signaling pathway [21]	Lipolysis and immune-signaling interface
ELOVL2	ELOVL2 can maintain the stability and integrity of membrane structures by promoting the synthesis of LC-PUFA, and promotes the EGFR signaling [22]	Elongation

*Abbreviations*: *FASN* Fatty acid synthase, *ZDHHC* zinc-finger and DHHC motif-containing, *FABP7* Fatty acid binding protein 7, *SCD1* Stearoyl-CoA desaturase 1, *DGAT1* Diacylglycerol acyltransferase 1, *MAGL* Monoacylglycerol lipase, *ELOVL2* Elongase of Very Long Chain Fatty Acids 2, *UFAs* unsaturated fatty acids, *ER* endoplasmic reticulum, *STAT1* signal transducer and activator of transcription 1, *ICAM1* intercellular adhesion molecule 1, *GSCs* glioblastoma stem cells, *ACLY* ATP citrate lyase, *nLDs* nuclear lipid droplets, *PML-NBs* promyelocytic leukemia nuclear bodies, *RXRα* retinoid X receptor α, *D-2HG* D-2-hydroxyglutarate, *MUFA* monounsaturated fatty acids, *LDs* lipid droplets, *PGE*_*2*_ prostaglandin E₂, *2-AG* 2-arachidonoylglycerol, *LC-PUFA* long-chain polyunsaturated fatty acids, *EGFR* epidermal growth factor receptor

### FASN

Fatty acid synthase (FASN) serves as a key enzyme in the fatty acid de novo synthesis pathway. Its main function is to catalyze acetyl-CoA and malonyl-CoA through a reaction cycle involving condensation, reduction, dehydration, and re-reduction to generate palmitic acid. This biosynthetic process is often abnormally upregulated in tumor cells, leading to the massive accumulation of palmitic acid. FASN promotes tumor cell malignancy mainly by regulating key biological processes [23–28]. For example, during hepatocarcinogenesis, zinc-finger and DHHC motif-containing 20 (ZDHHC20) can mediate the S-palmitoylation of FASN. This post-translational modification inhibits the degradation of FASN, improves its protein stability, and ultimately causes the intracellular accumulation of FAs [29]. Moreover, by catalyzing the synthesis of palmitic acid, which is required for palmitoylation, FASN further enhances the S-palmitoylation of Phosphoglycerate Dehydrogenase (PHGDH) and FASN itself, ultimately facilitating the acquisition of chemoresistance in triple-negative breast cancer (TNBC) [30]. Notably, FASN expression has been shown to be associated with the World Health Organization (WHO) grade of gliomas. This observation has further prompted researchers to probe its specific function in GBM.

The massive accumulation of palmitic acid induced by FASN overexpression provides abundant precursor molecules for downstream reactions, including desaturation and elongation. In the radiotherapy-based treatment of GBM, radiotherapy (RT)-induced DNA damage interferes with the normal metabolism of GBM cells, thereby impairing endoplasmic reticulum (ER) function and inducing ER stress, which ultimately leads to tumor cell elimination. Freitas-Cortez et al. demonstrated that RT induces lipid metabolic remodeling in GBM cells, leading to marked accumulation of unsaturated fatty acids (UFAs) [12]. UFAs can function not only as metabolic signals that alleviate ER stress and protect tumor cells from apoptosis, but also as an alternative energy source supporting GBM cell survival. Moreover, the post-RT accumulation of UFAs facilitates the formation of lipid droplets (LDs). LDs serve as the major site for the synthesis of prostaglandin E₂ (PGE₂). PGE₂ plays a key role in tumor cell proliferation, cancer stemness maintenance, and immunosuppression [31–33]. This finding indirectly indicates that FASN overexpression provides an essential prerequisite for tumor cells to produce adequate UFAs. Consequently, targeting FASN-mediated fatty acid synthesis is expected to improve RT-induced metabolic reprogramming in GBM.

In addition to its role in fatty acid metabolism, FASN has recently been reported to participate in signaling events within glioma cells, which may subsequently reshape the glioma TME. In GBM, the TME comprises a highly complex cellular and non-cellular network [34]. Cytotoxic T lymphocytes (CTLs), macrophages, and dendritic cells are important immune components of the GBM TME; however, these cells commonly become exhausted or functionally impaired, which promotes immunosuppression and supports GBM progression and drug resistance [35, 36]. Tumor-associated macrophages (TAMs), which primarily include microglia and monocyte-derived macrophages, constitute the most abundant immune cell subset within the GBM TME. The majority of these cells are polarized toward an anti-inflammatory (M2) phenotype, thereby promoting the growth of GBM [37]. In addition, macrophage-derived anti-inflammatory cytokines can facilitate the activation and functional activity of immunosuppressive cell populations, ultimately contributing to GBM progression [38]. According to Zhang et al., the FASN/STAT1 signaling axis regulates ICAM1 secretion, which is directly linked to the migration of microglia. At the mechanistic level, FASN-induced STAT1 phosphorylation in glioma cells leads to elevated soluble ICAM1 expression and secretion, which subsequently attracts microglia to GBM cells and strengthens the immunosuppressive TME. These findings on the FASN/STAT1/ICAM1/microglia axis imply that GBM patients characterized by abundant macrophage infiltration may respond more favorably to FASN inhibitors, which could suppress tumor cell proliferation while limiting macrophage recruitment [13].

Under hypoxia-induced metabolic stress, FASN-mediated accumulation of fatty acids can supply energy to tumor cells via fatty acid β-oxidation. In addition, hypoxia represents a key trigger of tumor angiogenesis and plays a crucial role in promoting resistance to antiangiogenic therapies [39, 40]. William Kelly’s research demonstrated that the combination of FASN inhibitors and bevacizumab can further enhance the therapeutic effect of bevacizumab [41].

Taken together, FASN appears to couple de novo FA synthesis with therapy adaptation and immune remodeling in GBM, although its subtype specificity and therapeutic window in the brain remain insufficiently defined.

### ZDHHC family and protein S-palmitoylation

As a reversible post-translational modification (PTM), protein S-acylation involves the thioester bond-mediated covalent linkage of medium- or long-chain fatty acids to selected cytosolic cysteine residues [42]. By increasing protein hydrophobicity, this lipid modification promotes membrane association and consequently regulates protein trafficking, stability, subcellular localization, and signal transduction [43–45]. Because palmitate is the most commonly attached fatty acid, S-acylation is often referred to as S-palmitoylation [42]. The major enzymes responsible for this process are ZDHHC protein acyltransferases, which contain a conserved DHHC cysteine-rich catalytic domain [42]. The human genome contains 23 genes encoding ZDHHC family members, with almost all known S-acylating enzymes showing expression in the brain [46]. The reversibility of S-acylation enables it to serve as a dynamic molecular switch, in a manner comparable to phosphorylation, acetylation, and ubiquitylation [42].

In their 2025 Neuro-Oncology study, Wang et al. reported that ZDHHC15-mediated palmitoylation enhances O-glycosylation, homodimerization, and activation of cellular mesenchymal-epithelial transition factor (c-MET), thereby preserving the stemness of glioblastoma stem cells (GSCs). Treatment with the FASN inhibitor TVB-3166 effectively blocked this process, thereby inhibiting intracranial tumor progression and prolonging survival in tumor-bearing mice [14].

As an increasingly recognized PTM, protein S-palmitoylation has been extensively investigated in a wide range of malignancies beyond glioma. For instance, ZDHHC20 promotes S-palmitoylation of YTH domain-containing family protein 3 (YTHDF3) at Cys474, which protects YTHDF3 from degradation, stabilizes MYC messenger RNA (mRNA), and ultimately supports the malignant behavior of pancreatic ductal adenocarcinoma cells [47]. In addition, through direct interaction with missense mutant p53 (mutp53), FASN enhances mutp53 palmitoylation, which reduces its ubiquitination-dependent degradation and consequently promotes mutant p53 accumulation and gain-of-function (GOF) in cancer [48]. Moreover, zDHHC8 promotes the palmitoylation of glutathione peroxidase 4 (GPX4), which strengthens ferroptosis resistance in tumor cells and consequently reduces CD8 + cytotoxic T cell-mediated ferroptosis, leading to weakened antitumor immunity [49].

Collectively, these findings suggest that fatty acid-dependent protein palmitoylation can modulate therapeutic responses and antitumor immunity in cancer. Taken together, protein palmitoylation, as a fatty acid metabolism-associated PTM, represents a promising and underexplored area of investigation in glioma.

### FABP7

FABP7 is also termed brain-type fatty acid binding protein (B-FABP) or brain lipid binding protein (BLBP). Its main function is to bind and solubilize long-chain FAs (especially long-chain UFAs), promote their intracellular uptake and transport, and thereby regulate lipid metabolism. In GBM, high FABP7 expression is an independent poor prognostic factor [50].

Through a comprehensive multi-database analysis, Hou et al. showed that FABP7 could independently predict prognosis in glioma patients. Significant associations have been observed between FABP7 expression and several clinicopathological features, including patient age, tumor grade, chemotherapy status, 1p19q codeletion status, and IDH1 mutation status. FABP7 may further promote tumor progression through its involvement in tumor angiogenesis [50].

Nuclear acetyl-CoA is generated through the pathway mediated by ATP citrate lyase (ACLY). It is an essential molecule for signal transduction and epigenetic regulation that can act as an acetyl donor for histone lysine acetylation [51, 52]. Yoshiteru Kagawa et al.'s study showed that FABP7 can regulate the level of nuclear acetyl-CoA by interacting with ACLY in GBM, which subsequently increases the level of histone H3 lysine 27 acetylation (H3K27ac) in the caveolin-1 promoter region. Through this epigenetic regulation, FABP7 promotes caveolin-1 expression, thereby impacting the formation and function of caveolae. Ultimately, FABP7 is involved in GBM cells' proliferation to regulate the biological characteristics of the tumor [15].

Moreover, the study by Banlanjo Abdulaziz Umaru et al. revealed that binding between FABP7 and intracellular long-chain FAs, particularly oleic acid (OA), drives the translocation of FABP7 into the nucleus and induces the generation of nuclear lipid droplets (nLDs) colocalized with PML-NBs. The study further confirmed that the FABP7-OA complex can directly drive tumor cell proliferation by epigenetically regulating the secretion process of proteins encoded by proliferation-related genes [16].

Notably, in glioblastoma stem cells (GSCs), FABP7 can also affect the migration ability of GSCs by regulating cellular signaling transduction. Liu et al. reported in Neuro-Oncology (2023) that free PUFAs in GSCs can bind to FABP7 and be transported to the nucleus. Nuclear PUFAs subsequently bind to and activate the nuclear receptor RXRα, which in turn upregulates the expression of EMT markers and stemness markers, inducing the stationary-to-migratory phenotypic transition of GSCs, and ultimately promoting the progression of gliomas [17, 53].

In summary, FABP7 represents more than a lipid chaperone in glioma. It integrates FA transport with nuclear signaling and stemness. FABP7 inhibitors may provide a novel targeted strategy for GBM treatment, although their clinical translation remains to be established.

### Other targets

FASN, the ZDHHC family, and FABP7 respectively represent alterations in fatty acid synthesis, utilization, and transport in glioma, and currently constitute some of the most extensively studied and widely recognized targets in this field. In addition to rewiring fatty acid synthesis, utilization and transport to influence therapeutic responses, tumor cells may also alter fatty acid storage, lipolysis, and elongation to contribute to temozolomide resistance and radioresistance. Nevertheless, current knowledge of these targets is largely supported by preclinical data, and their applicability to human patients still needs to be validated through further investigation.

#### SCD1

The synthesis of MUFA, such as OA, is regulated by SCD1. SCD1 has been widely reported to function as a regulator of cancer progression, cell survival, differentiation, and malignant transformation in human cancers [54]. In IDH-mutant glioma cells, mutant IDH1 (IDH1mut) gains neomorphic enzymatic activity and uses nicotinamide adenine dinucleotide phosphate (NADPH) to produce D-2-hydroxyglutarate (D-2HG) [55, 56]. Adrian Lita et al.’s study demonstrated that D-2HG can significantly upregulate the expression level of intracellular SCD1. Furthermore, the mRNA level of SCD1 is the highest in oligodendroglioma tissues. Upregulated SCD1 expression further results in increased levels of MUFAs and associated phospholipids, which directly cause the expansion of the Golgi apparatus and ER in IDH1mut glioma cells. By contrast, IDH1 wild-type (IDH1WT) GBM cells do not have the MUFAs upregulation and phospholipid imbalance mentioned above, and their Golgi apparatus and ER both display normal morphology [18]. Adrian et al. also mentioned that when inducing the phospholipid imbalance to shift toward a further increase in MUFA, IDH1mut cells are more sensitive to MUFA-induced apoptosis compared with IDH1WT cells. These results may guide the future screening and selection of potential therapeutic targets for IDH-mutant tumors [18]. In addition, the study by Dai et al. showed that TMZ-resistant GBM cells exhibit marked upregulation of SCD1. By activating the Akt/glycogen synthase kinase 3 β (GSK3β)/β-catenin signaling pathway, elevated SCD1 expression directly contributes to the growth and migration of GBM cells [19]. These observations suggest that SCD1, together with the functional pathways it regulates, may play a critical role in TMZ resistance. Accordingly, targeting SCD1 alone or in combination with Akt signaling inhibition could be a potential strategy to overcome TMZ resistance in gliomas.

#### DGAT1

Diacylglycerol acyltransferase 1 (DGAT1) is a transmembrane protein residing in the ER that serves as a key enzyme catalyzing the esterification of acyl-CoA with DG to produce triacylglycerol (TG). Subsequently, TGs participate in the formation of lipoprotein precursor structures, which emerge from the ER into the cytosol and progressively mature into LDs [57–59].

As lipid-storage organelles, LDs are widely present in GBM, whereas they are generally not detected in normal brain tissue [60, 61]. The distinct distribution of LDs implies abnormal activation of DGAT1-dependent lipid metabolism in GBM, in contrast to the low DGAT1 expression observed in normal brain tissue. TG constitutes the major component of LDs. When nutrients are limited, tumor cells rapidly mobilize LD-stored lipids through lipolysis, producing free fatty acids (FFAs) that can be utilized for membrane lipid synthesis and energy production to sustain cell survival [62, 63]. In GBM, excess FAs are esterified into TG and deposited in LDs, thereby helping maintain lipid homeostasis [64]. Cheng et al. showed that inhibition of DGAT1-mediated fatty acid storage triggers pronounced oxidative stress and disturbs lipid homeostasis in GBM cells. However, this effect has no impact on normal brain tissues with low DGAT1 expression. When DGAT1 is inhibited, lipid homeostasis is disrupted, causing excessive FFAs to be transported into mitochondria for β-oxidation. Excessive reactive oxygen species (ROS) are generated during this process, resulting in mitochondrial damage and cytochrome c release. These events eventually trigger tumor cell apoptosis and markedly suppress GBM growth [64].

Furthermore, studies have revealed that DGAT1 expression is upregulated after ionizing radiation (IR), suggesting that it is potentially associated with GBM radioresistance. When DGAT1 is suppressed through post-transcriptional gene silencing (PTGS) using short hairpin RNA (shRNA) or microRNA (miRNA), GBM cells exhibit significantly reduced radioresistance [20]. Diacylglycerol kinases (DGKs) comprise an enzyme family responsible for catalyzing the conversion of DG into phosphatidic acid (PA). They can antagonize DGAT1 activity by decreasing the level of DG in the cell membrane and competing with DGAT1 for the substrate. Hyunkoo Kang et al.'s study demonstrated that IR can simultaneously induce the upregulation of DGAT1 expression and the downregulation of diacylglycerol kinase B (DGKB) expression, and these two pathways have a synergistic effect on the development of GBM radioresistance [20]. Notably, the use of cladribine can reverse this process. On the one hand, it upregulates the expression of DGKB, and on the other hand, it inhibits the expression of DGAT1, ultimately reducing radioresistance and restoring radiosensitivity in GBM cells. This finding suggests that targeting lipid homeostasis may be a potential strategy for overcoming GBM radioresistance.

Up to now, studies focused on DGAT1-mediated tumor metabolic reprogramming have been increasing. Besides GBM, aberrant DGAT-dependent LDs synthesis has also been detected in hepatocellular carcinoma (HCC) and renal cell carcinoma (RCC) [64, 65]. Therefore, the combination of DGAT1-targeted inhibition and standard GBM therapy may represent a promising approach for addressing resistance to conventional treatment.

#### MsAGL

Encoded by the MGLL gene, Monoacylglycerol lipase (MAGL) functions as a major member of the lipolytic enzyme family and is mainly responsible for hydrolyzing monoacylglycerols to generate glycerol and FFAs [66]. In aggressive cancer cells, MAGL high expression is associated with tumor malignancy. From the perspective of downstream regulatory pathways, the effects of MAGL on cancer cells are mainly reflected in two aspects. First, it hydrolyzes monoacylglycerols to produce FFAs, directly modulating the lipid metabolic homeostasis of tumor cells to meet the metabolic requirements for malignant proliferation. Second, it can also hydrolyze 2-arachidonoylglycerol (2-AG) to AA, and AA is subsequently converted into PGE₂ [67], which participates in signaling pathway regulation. According to Yin et al., MAGL regulates PGE₂ production, leading to increased intracellular β-catenin accumulation, which subsequently supports GSC self-renewal and tumorigenicity [21]. This study also revealed that MAGL can further modulate the polarization phenotype of macrophages in the TME of glioma. Higher MAGL expression is accompanied by increased M2-type polarization of TAMs. Knockdown of MAGL can significantly increase the polarization level of M1-type TAMs and effectively reverse the immunosuppressive TME [21]. Yin et al. further clarified the upstream regulatory mechanism and found that arsenite-resistance gene 2 (ARS2) can activate MAGL transcription. Specifically, ARS2 binds to the regulatory region of the MGLL gene and directly increases MAGL expression at the transcriptional level, forming an upstream ARS2-MAGL regulatory axis [21].

#### ELOVL2

The elongation of LC-PUFAs is primarily catalyzed by ELOVL2, a key enzyme in LC-PUFAs synthesis. Compared with normal brain tissue, GBM tissue not only has significantly higher levels of FFAs and LC-PUFAs, but also exhibits distinct alterations in phospholipid composition [68]. This observation implies aberrant activation of ELOVL2-mediated LC-PUFA synthesis in GBM. Using a super-enhancer profiling approach, Ryan C. Gimple et al. found that GBM cells and GSCs exhibit elevated ELOVL2 expression driven by a GSC-specific non-coding regulatory region, ultimately promoting LC-PUFA production [22]. Mechanistically, LC-PUFAs help maintain cell membrane stability and integrity, thereby providing essential support for effective transmission of the EGFR signaling pathway. Based on this, Ryan C. Gimple proposed that ELOVL2 and the EGFR pathway may have a synergistic effect in gliomas. ELOVL2 promotes LC-PUFAs production, thereby maintaining the structural stability of cell membranes and supporting normal cellular functions. At the same time, this process enhances EGFR signaling transduction and ultimately drives tumor progression [22]. EGFR has become a key target for numerous preclinical studies and clinical trials in the field of GBM treatment [69–71]. However, existing EGFR-targeting agents have consistently failed to effectively extend patients' survival, suggesting that there are therapeutic limitations in the single-targeting of EGFR. Study data from Ryan C. Gimple's team demonstrate that the combination therapeutic strategy of simultaneously targeting the EGFR signaling pathway and the LC-PUFA synthesis process may overcome the limitations of single-targeting and significantly improve therapeutic efficacy [22]. This finding provides a new direction for combination therapy in GBM treatment and has significant clinical translational value.

### Clinical trials targeting fatty acid metabolism in glioma

Although drugs targeting glioma metabolism are still not approved for clinical use, several clinical trials targeting fatty acid metabolism in glioma have been carried out (Table 2). The phase II clinical trial NCT03032484 investigated TVB-2640, a FASN inhibitor, in combination with bevacizumab and reported a PFS6 of 31.4% in the combination therapy group. This value was significantly higher than the historical PFS6 of 16% observed with bevacizumab monotherapy (P = 0.008). In patients with recurrent high-grade astrocytoma, this phase II trial showed favorable tolerability of oral TVB-2640 and revealed a significant improvement in progression-free survival when TVB-2640 was combined with bevacizumab [41]. Currently, the positive outcomes achieved by this combination therapy regimen provide strong support for further conducting a phase III clinical trial. Additionally, another phase III clinical trial (NCT05118776) evaluating an FASN inhibitor in combination with bevacizumab for the treatment of recurrent GBM is still ongoing. To date, the relevant study results of this trial have not been published.

**Table 2:** Clinical trials targeting fatty acid metabolism in glioma

Drugs	Conditions	Target	Phases	NCT Number
TVB-2640	Astrocytoma	FASN	PHASE2	NCT03032484
ASC40	rGBM	FASN	PHASE3	NCT05118776
pioglitazone	GBM	PPAR-γ	PHASE1	NCT01151670
Pioglitazone + Rofecoxib	GBM	PPAR-γ、COX-2	PHASE2	

*Abbreviations*: *rGBM* recurrent glioblastoma, *COX-2* cyclooxygenase 2

At present, specific agents targeting fatty acid metabolism have not been approved for clinical use; however, some approved non-oncological drugs capable of modulating these pathways are being explored for their potential roles in cancer treatment (Table 2). Specifically, the FASN inhibitor orlistat, originally approved for obesity, has demonstrated clear antitumor activity in preclinical studies. Mechanistically, it exerts antitumor effects by promoting tumor cell apoptosis, reducing programmed death-ligand 1 (PD-L1) expression, and blocking the signal transducer and activator of transcription 3 (STAT3)/nuclear factor kappa B (NF-κB) signaling pathway, a major regulator of tumor proliferation and immunosuppression [72–74]. In addition, the combined use of pioglitazone, a peroxisome proliferator-activated receptor γ (PPAR-γ) agonist, rofecoxib, a cyclooxygenase-2 (COX-2) inhibitor, and TMZ was investigated in a phase II clinical trial involving glioma patients. Results showed that although this regimen did not significantly improve patients' mOS, 2 patients achieved a partial response (PR) lasting for 32–44 months [75], suggesting that this combination strategy may have potential efficacy for a specific subset of patients.

## Conclusions

### Current understanding

Fatty acid metabolism in glioma has recently attracted considerable academic attention. FA metabolic rewiring can influence the malignant progression and treatment outcomes of gliomas in multiple ways. On the one hand, aberrant fatty acid metabolism can induce resistance to radiotherapy and chemotherapy. On the other hand, alterations in fatty acid metabolism can impair antitumor immune responses by remodeling the tumor microenvironment and/or reducing the vulnerability of tumor cells to CD8 + T cell-mediated killing.

### Future directions

Based on the above mechanisms, future research could focus on the clinical translation of the fatty acid metabolic reprogramming in glioma. Combination therapy with fatty acid metabolic intervention drugs and other treatments may overcome therapeutic resistance. For instance, FASN inhibitor-based radiosensitization may represent a potential approach to reduce malignant recurrence and improve clinical outcomes. In addition, MAGL inhibitors may enhance the response to immunotherapy by reshaping the immunosuppressive TME. Given the potential crosstalk between EGFR signaling and ELOVL2-mediated lipid metabolism, dual inhibition of these targets may be superior to EGFR-targeted therapy alone. Additionally, because protein palmitoylation enhances the affinity of proteins for biomembranes, it may influence processes such as nuclear trafficking and vesicular transport. Therefore, palmitoylation may provide an important mechanistic bridge linking fatty acid metabolic rewiring to DNA damage repair or TME remodeling in glioma. Collectively, we propose that palmitoylation represents a highly promising direction for future research in glioma.

### Challenges and limitations

We propose that the core limitation of metabolic therapy is that, beyond targeting tumor cells, it may also affect the normal metabolism of other cells, such as neurons, natural killer (NK) cells, T cells, and macrophages, which may result in off-target effects. Consequently, it is a challenge for future studies to screen more specific targets based on the unique metabolic features of tumor cells. Furthermore, as a specialized vascular interface, the blood–brain barrier (BBB) protects and nourishes brain neurons and glia while simultaneously limiting the delivery of therapeutic agents. The BBB therefore represents a major barrier to the identification and development of small-molecule therapeutics. Gao et al. recently reported an intracalvariosseous injection-based strategy in which drug-loaded nanoparticles exploit calvarial immune cells and their skull–meninges microchannel-mediated migration to bypass the BBB and achieve central nervous system drug delivery [76]. Furthermore, intraventricular administration via an Ommaya reservoir may offer a practical strategy to overcome the limited BBB permeability of many therapeutic agents and thereby improve intracranial drug delivery.

In addition to directly targeting the metabolism of tumor cells, another direction of investigation focuses on regulating the metabolism of immune cells [77]. Enhancing the activity of pro-inflammatory immune cells (e.g., effector T cells) by metabolic intervention can remodel immunosuppressive TME and promote anti-tumor immune responses. However, research on the metabolic regulation of immune cells in GBM remains limited, partly because the number of tumor-infiltrating immune cells in the GBM microenvironment is relatively low. Therefore, substantial efforts and further breakthroughs are still needed in future studies.

## Data Availability

No datasets were generated or analysed during the current study.

## Abbreviations

GBM: Glioblastoma
IDH: Isocitrate dehydrogenase
mOS: Median overall survival
TMZ: Temozolomide
FAs: Fatty acids
DG: Diacylglycerol
PUFAs: Polyunsaturated fatty acids
AA: Arachidonic acid
COX: Cyclooxygenase
PGs: Prostaglandins
TXA2: Thromboxane A2
LOX: Lipoxygenase
TME: Tumor microenvironment
FASN: Fatty acid synthase
EMT: Epithelial-mesenchymal transition
PHGDH: Phosphoglycerate dehydrogenase
TNBC: Triple-negative breast cancer
ER: Endoplasmic reticulum
UFAs: Unsaturated fatty acids
LDs: Lipid droplets
PGE₂: Prostaglandin E₂
CTLs: Cytotoxic T lymphocytes
TAMs: Tumor-associated macrophages
Tregs: Regulatory T cells
MDSCs: Myeloid-derived suppressor cells
ICAM1: Intercellular adhesion molecule 1
ZDHHC: Zinc-finger and DHHC motif-containing
PTM: Post-translational modification
FABP7: Fatty acid binding protein 7
FABP: Fatty acid binding protein
B-FABP: Brain-type fatty acid binding protein
BLBP: Brain lipid binding protein
CGGA: Chinese glioma genome atlas
TCGA: The cancer genome atlas
GEO: Gene expression omnibus
ACLY: ATP citrate lyase
H3K27ac: H3 lysine 27 acetylation
OA: Oleic acid
nLDs: Nuclear lipid droplets
PML-NBs: Promyelocytic leukemia nuclear bodies
GSCs: Glioblastoma stem cells
RXRα: Retinoid X receptor α
SCD1: Stearoyl-coa desaturase 1
MUFA: Monounsaturated fatty acids
IDH1mut: Mutant IDH1
NADPH: Nicotinamide adenine dinucleotide phosphate
D-2HG: D-2-hydroxyglutarate
IDH1WT: IDH1 wild-type
DGAT1: Diacylglycerol acyltransferase 1
TG: Triacylglycerol
FFAs: Free fatty acids
ROS: Reactive oxygen species
IR: Ionizing radiation
PTGS: Post-transcriptional gene silencing
shRNA: Short hairpin RNA
miRNA: Microrna
DGKs: Diacylglycerol kinases
PA: Phosphatidic acid
DGKB: Diacylglycerol kinase B
HCC: Hepatocellular carcinoma
RCC: Renal cell carcinoma
MAGL: Monoacylglycerol lipase
2-AG: 2-Arachidonoylglycerol
ARS2: Arsenite-resistance gene 2
ELOVL2: Elongase of Very Long Chain Fatty Acids 2
LC-PUFAs: Long-chain polyunsaturated fatty acids
EGFR: Epidermal growth factor receptor
PFS6: The 6-month progression-free survival rate
PD-L1: Programmed death-ligand 1
STAT3: Signal transducer and activator of transcription 3
NF-κB: Nuclear factor kappa B
PPAR-γ: Peroxisome proliferator-activated receptor γ
COX-2: Cyclooxygenase-2
PR: Partial response
NK: Natural killer
FA: Fatty acid
ATP: Adenosine triphosphate
CDP-DG: Cytidine diphosphate-diacylglycerol
WHO: World health organization
RT: Radiotherapy
c-MET: Cellular mesenchymal-epithelial transition factor
YTHDF3: YTH domain-containing family protein 3
GOF: Gain-of-function
GPX4: Glutathione peroxidase 4
mRNA: Messenger RNA
BBB: Blood-brain barrier
AKT: AKT serine/threonine kinase
GSK3β: Glycogen synthase kinase 3 β
